# Barriers and facilitators to online medical and nursing education during the COVID-19 pandemic: perspectives from international students from low- and middle-income countries and their teaching staff

**DOI:** 10.1186/s12960-021-00609-9

**Published:** 2021-05-12

**Authors:** Wen Li, Robyn Gillies, Mingyu He, Changhao Wu, Shenjun Liu, Zheng Gong, Hong Sun

**Affiliations:** 1 School of International Education, Xuzhou Medical University, No. 209 of Tongshan Road, Yunlong, Xuzhou, 221004 China; 2 School of Education, The University of Queensland, Brisbane, 4072 Australia; 3 School of Biosciences and Medicine, Faculty of Health and Medical Sciences, University of Surrey, Guildford, GU2 7XH Surrey UK; 4 School of Public Affairs and Governance, Silliman University, 6200 Dumaguete, Philippines

**Keywords:** Assessment, COVID-19, International medical students, International nursing students, Low- and middle-income countries, Online learning, Satisfaction

## Abstract

**Background:**

The COVID-19 pandemic posed a huge challenge to the education systems worldwide, forcing many countries to provisionally close educational institutions and deliver courses fully online. The aim of this study was to explore the quality of the online education in China for international medical and nursing students from low- and middle-income countries (LMICs) as well as the factors that influenced their satisfaction with online education during the COVID-19 pandemic.

**Methods:**

Questionnaires were developed and administered to 316 international medical and nursing students and 120 teachers at a university in China. The Chi-square test was used to detect the influence of participants’ personal characteristics on their satisfaction with online education. The Kruskal–Wallis rank-sum test was employed to identify the negative and positive factors influencing the online education satisfaction. A binary logistic regression model was performed for multiple-factor analysis to determine the association of the different categories of influential factors—crisis-, learner-, instructor-, and course-related categories, with the online education satisfaction.

**Results:**

Overall, 230 students (response rate 72.8%) and 95 teachers (response rate 79.2%) completed the survey. It was found that 36.5% of students and 61.1% of teachers were satisfied with the online education. Teachers’ professional title, students’ year of study, continent of origin and location of current residence significantly influenced the online education satisfaction. The most influential barrier for students was the severity of the COVID-19 situation and for teachers it was the sense of distance. The most influential facilitating factor for students was a well-accomplished course assignment and for teachers it was the successful administration of the online courses.

**Conclusions:**

Several key factors have been identified that affected the attitudes of international health science students from LMICs and their teachers towards online education in China during the COVID-19 pandemic. To improve the online education outcome, medical schools are advised to promote the facilitating factors and cope with the barriers, by providing support for students and teaching faculties to deal with the anxiety caused by the pandemic, caring for the state of mind of in-China students away from home, maintaining the engagement of out-China students studying from afar and enhancing collaborations with overseas institutions to create practice opportunities at students’ local places.

## Background

The coronavirus disease 2019 (COVID-19) pandemic undoubtedly posed a huge challenge to the education systems worldwide, disrupting the normal teaching and learning trajectories [1]. As per UNESCO [2], over 100 countries had provisionally closed their educational institutions nationwide to contain the spread of COVID-19, and some countries had implemented localised school closures in large regions.

The COVID-19 virus broke out during students’ winter vacation in China, and thus the coming spring semester faced the risk of suspension due to the national policy of temporary school closures. To address students’ learning needs, the Chinese authorities responded by advising the education institutions at different learning levels to initiate fully online education starting from February, 2020 [3, 4]. At present, there are more than 68,000 international students studying medicine and nursing in about 100 of China’s institutions, mainly from low- and middle-income countries (LMICs) in Asia and Africa [5–7]. They have also been receiving online teaching during the COVID-19 crisis [8].

Although online education is well recognised and documented as a promising and effective mode for teaching undergraduate medical and nursing students [9, 10], it was a different story to conduct fully online courses during the COVID-19 crisis, as the online courses’ function and people’s mental states can be very different from those during ordinary times [11]. More importantly, as for the international students who were currently learning distantly from their resources-limited homelands, the institutional readiness in technological and infrastructural supplies is not always present [12], which makes cross-national online education more challenging.

There have been several studies which assess online medical and nursing education amid the pandemic from Jordan [13], India [14], Saudi Arabia [15] and China [16], providing useful feedback on the online education for domestic students. In fact, as the circumstances began to stabilise, domestic students have started to progressively return to campus and face-to-face teaching has been gradually resumed. By comparison, for international students, online learning will remain as the main instructing modality for the foreseeable future [17], as many of them are experiencing difficulties returning to campus due to the international travel ban. Therefore, it is imperative and urgent to understand the landscape of online courses for this group of students, so as to monitor and improve the quality of the international courses. However, to the best of our knowledge, studies discussing the online education for international medical and nursing students from LMICs are unavailable.

Learner satisfaction and teacher satisfaction, which relate to their attitudes towards the education experiences and the achieved education outcomes [18, 19], are among the “five quality pillars” in the quality framework of the Online Learning Consortium [20] and are thus highly predictive of the quality and outcome of the online courses [21]. Therefore, exploration of learners’ and teachers’ online education satisfaction and the influential factors can provide important guidance and reference for the improvement of the online education outcomes.

To understand the quality and influential factors of online health sciences education in China for international students during the COVID-19 pandemic, we surveyed a university in China (a provincial medical university representing the overwhelming majority of China’s medical universities), with the aim to investigate the following aspects in our study:

1. attitudes of this university’s international medical students (IMSs), international nursing students (INSs) and their teachers towards the online education experiences during the COVID-19 crisis,
2. personal characteristics affecting students’ and teachers’ online education satisfaction,
3. factors affecting students’ and teachers’ online education satisfaction,
4. differences in the influential factors for students staying inside China and those outside China, and
5. the influence of the COVID-19 crisis on online education.

## Methods

### Setting and participants

This questionnaire-based study was conducted between the 14th April and 23rd April, 2020, among the IMSs from 1st to 5th year, the INSs from 1st to 3rd year and their teachers at Xuzhou Medical University (XZMU), China. This university began to deliver online courses to its international students from the 24th of February, 2020, and up to the time of the surveys, students had been learning online for nearly 2 months.

The online learning package tailored to the IMSs and INSs is blended with synchronised and asynchronised modes, delivering theoretical knowledge through a combination of real-time live lessons, recorded video lessons, required readings, compulsory assignments, and interactive activities (class discussions and communications about assignments), by means of live streaming platforms, video-uploading websites, emails, and social networking software. The learning goals and key points are provided alongside the course materials.

This study was approved by the Ethics Committee of Xuzhou Medical University.

### Questionnaire design

There were two questionnaires, one for the international students and one for their teachers. The questionnaires were developed following 3 steps [22].

Step 1: This step was to collect information on the influential factors for the online education. Data were collected from international course teachers (individual views), international course directors (comprehensive views), international students (individual views) and international student monitors (comprehensive views). We sent emails to the course teachers and left messages in the student online-chatting class groups to kindly request their individual feedback on the factors that influenced their online education. Twenty-eight teachers and 34 students responded to our request by sending emails or leaving messages. Ten course directors were contacted by phone for a more comprehensive perspective, and notes were kept during the interview process. In addition, we invited 14 student monitors to participate in an online focus group discussion via the online chatting tool, where communications took place by typing words or sending voicemails. As student monitors were the coordinators between teachers and class members, they were more involved in and more familiar with each link during the whole online education process. During the focus group discussion, the student monitors were able to provide feedback from a more collective and general point of view through in-depth communications with us and between themselves. Responses were then summarised and transcribed into a Word document and analysed thematically.

Step 2: After an intensive literature review surrounding the topic of online education, more possible factors influencing online learning were added to the questionnaires to ensure the comprehensiveness [10, 23]. The questions regarding the factors related to online education, based on the typology proposed by Zheng et al. [24], were then divided into learner-related questions, instructor-related questions, course-related questions, and additionally, crisis-related questions to fit for the purpose of our study. Then the structure of the questionnaires for students and teachers was settled and the draft version of the questionnaires was formulated.

Step 3: A panel of 4 experts in the field of health education and online education were invited to evaluate the content of the questionnaires with regard to aspects of the rationality of the items, overlapping or similar items, and the readability and interpretability of the items. The questionnaires were revised according to the experts’ comments and finalised. Then we invited 2 students monitors and 2 class members to pilot-test the students’ questionnaire, 2 course teachers and 2 course directors to pilot-test the teachers’ questionnaire. They all provided very positive feedback for the design of the questionnaires.

The questionnaires for students and teachers had a similar format and both contained 3 sections. The first section collected the participants’ basic information, such as age, gender and facilities used for online learning/teaching. The students’ questionnaire also included grade, nationality and current residence, and the teachers’ questionnaire included professional title, teaching experience, work type, place for conducting the online teaching, and teaching subject(s). The second section asked about the participants’ perceptions of their online education experiences and expectations, such as the general satisfaction of the online education, acceptance of this new modality, preference of future education style, as well as the time and dedication spent on online learning/teaching. The third section included five-point Likert-scale questions about the factors influencing the participants’ satisfaction with their online education experiences, with 43 items on the students’ side and 39 items on the teachers’ side.

### Data collection and analysis

Electronic questionnaires were employed. The questionnaire for the students was in English and that for the teachers was in Chinese. In the questionnaires, it was clearly explained that the participation was completely voluntary and the aim was to investigate the international medical and nursing education. All participants gave written consent for their opinions to be published anonymously.

The data obtained from this study were analysed using IBM SPSS Statistics (version 24.0). Responses to the five-point Likert-scale questions ranged from “strongly disagree/very poor/very low/very little (1 point)” to “strongly agree/excellent/very high/very much (5 point)”. The Chi-square test was used to determine the influence of the characteristics of students/teachers on the education satisfaction and to compare the experiences and expectations of online education between students and teachers. The Kruskal–Wallis rank-sum test was performed to identify the positive and negative factors influencing students’ and teachers’ online education satisfaction. A binary logistic regression model was performed to compare the influence of factors in different categories on the online education satisfaction.

## Results

Information on the students’ characteristics is provided in Table 1. In total, 316 international students at the university who had been receiving an online education were invited to complete the questionnaire and 230 responded, giving a response rate of 72.8%. Among all the participating students, 130 (56.5%) were Asian students and 100 (43.5%) were African students. Most of the students (71.3%) used the mobile phone for online learning. During the online learning time, 77 (33.5%) were staying inside China, and 153 (66.5%) were staying outside China; to be specific, 147 (63.6%) were in their home countries.

**Table 1:** Students’ personal characteristics

Characteristics	Number (percentage)
*Gender*	
Male	82 (35.7%)
Female	148 (64.3%)
*Age*	
17–19	43 (18.7%)
20–22	129 (56.1%)
23–25	58 (25.2%)
*Major*	
MBBS	207 (90.0%)
Nursing	23 (10.0%)
*Year of study*	
1st year	52 (22.6%)
2nd year	50 (21.7%)
3rd year	55 (23.9%)
4th year	38 (16.5%)
5th year	35 (15.2%)
*Countries of origin*	
Asian countries^a^	130 (56.5%)
African countries^b^	100 (43.5%)
*Current residence*	
Inside China	77 (33.5%)
Outside China	153 (66.5%)
*Facilities used for online learning*	
Mobile phone	164(71.3%)
Computer/notebook	50 (21.7%)
Pad	16 (7.0%)
Total	230 (100%)

^a^Participants in our study were from 10 Asian countries, including Bangladesh, India, Lao PDR, Malaysia, Pakistan, Philippines, Sri Lanka, Syrian Arab Republic, Timor-Leste and Yemen, Rep

^b^Participants in our study were from 20 African countries, including Botswana, Comoros, Congo, Rep., Ethiopia, Gabon, Ghana, Kenya, Lesotho, Malawi, Morocco, Nigeria, Rwanda, Sierra Leone, Somalia, South Africa, Swaziland, Tanzania, Uganda, Zambia and Zimbabwe

Table 2 describes the teachers’ personal characteristics. A total of 120 teachers who had been teaching IMSs/INSs online courses were invited to participate in the study and 95 of them responded, translating to a response rate of 79.2%. About 48.4% of the responding teachers were lecturers or teaching assistants. Fifty-one (53.7%) taught basic subjects and 83 (87.4%) undertook work in addition to teaching. The majority of them (74.7%) had no online teaching experience and the bulk of the teachers (80.0%) taught at home.

**Table 2:** Teachers’ personal characteristics

Characteristics	Number (percentage)
*Gender*	
Male	40 (42.1%)
Female	55 (57.9%)
*Age*	
25–34	25 (26.3%)
35–44	56 (58.9%)
45–54	14 (14.7%)
*Professional title*	
Lecturer and below	46 (48.4%)
Associate professor	35 (36.8%)
Professor	14 (14.7%)
*Teaching subject type*	
Basic subjects	51 (53.7%)
Clinical subjects	44 (46.3%)
*Work type*	
Teaching and others	83 (87.4%)
Teaching only	12 (12.6%)
*Teaching experience*	
Less than 5 years	49 (51.6%)
5 years or more	46 (48.4%)
*Previous online teaching experience*	
Some	24 (25.3%)
None	71 (74.7%)
*Extra communications after class with students*	
Yes	70 (73.7%)
No	25 (26.3%)
*Place for conducting the online teaching*	
Home	76 (80.0%)
Work place	19 (20.0%)
Total	95 (100%)

### Personal characteristics affecting students’ and teachers’ online education satisfaction

Table 3 shows the influence of the students’ characteristics on their assessment of online education quality. For the purpose of discussion, students’ years of study were divided into basic years and clinical years and their countries of origin were grouped into Asian and African countries. Overall, 36.5% of the students were positive about the online learning. More students in their clinical years (72.5%) were negative about the online learning effect than those in their basic years (58.7%) (*P* < 0.05). A higher percentage of African students (73.0%) were dissatisfied with online learning compared to their Asian counterparts (56.2%) (*P* < 0.01). In addition, the percentage of the dissatisfied students inside China (80.5%) was higher than that of dissatisfied students outside China (54.9%) (*P* < 0.01).

**Table 3:** Students’ personal characteristics affecting their online education satisfaction (*n* = 230)

Characteristics	Satisfied with online education	Not satisfied with online education	*p*-value
	84 (36.5%)	146 (63.5%)	
*Gender*			
Male	33 (40.2%)	49 (59.8%)	.383
Female	51 (34.5%)	97 (65.5%)	
*Age*			
17–19	16 (37.2%)	27 (62.8%)	.404
20–22	51 (39.5%)	78 (60.5%)	
23–25	17 (29.3%)	41 (70.7%)	
*Major*			
MBBS	77 (37.2%)	130 (62.8%)	.523
Nursing	7 (30.4%)	16 (69.6%)	
*Year of study*			
Basic years^a^	62 (41.3%)	88 (58.7%)	.038
Clinical years^b^	22 (27.5%)	58 (72.5%)	
*Countries of origin*			
Asian countries	57 (43.8%)	73 (56.2%)	.009
African countries	27 (27.0%)	73 (73.0%)	
*Current residence*			
Inside China	15 (19.5%)	62 (80.5%)	.000
Outside China	69 (45.1%)	84 (54.9%)	

^a^1st-, 2nd- and 3rd-year MBBS students and 1st- and 2nd-year nursing students were grouped into basic years, who studied basic sciences subjects

^b^4th- and 5th-year MBBS students and 3rd-year nursing students were grouped into clinical years, who were engaged in clinical studies

The influence of the teachers’ characteristics on their assessment of online education quality is shown in Table 4. Overall, 61.1% of the teachers were satisfied with the online education effect. Professional title was identified as a significant characteristic associated with teachers’ satisfaction of online education (*P* < 0.01), while other characteristics were not. Pairwise comparison revealed that the percentage of the dissatisfied professors (71.4%) was significantly higher than that of dissatisfied associate professors (28.6%).

**Table 4:** Teachers’ personal characteristics affecting their online education satisfaction (*n* = 95)

Characteristics	Satisfied with online education	Not satisfied with online education	*p*-value
	58 (61.1%)	37 (38.9%)	
*Gender*			
Male	23 (57.5%)	17 (42.5%)	.545
Female	35 (63.6%)	20 (36.4%)	
*Age*			
25–34	19 (76.0%)	6 (24.0%)	.202
35–44	31 (55.4%)	25 (44.6%)	
45–54	8 (57.1%)	6 (42.9%)	
*Professional title*			
Lecturer and below	29 (63.0%)	17 (37.0%)	.002
Associate professor	25 (71.4%)	10 (28.6%)	
Professor	4 (28.6%)	10 (71.4%)	
*Teaching subject type*			
Basic subjects	33 (64.7%)	18 (35.3%)	.432
Clinical subjects	25 (56.8%)	19 (43.2%)	
*Work type*			
Teaching and others	52 (62.7%)	31 (37.3%)	.529
Teaching only	6 (50.0%)	6 (50.0%)	
*Teaching experience*			
Less than 5 years	32 (65.3%)	17 (34.7%)	.380
5 years or more	26 (56.5%)	20 (43.5%)	
*Previous online teaching experience*			
None	41 (57.7%)	30 (42.3%)	.256
Some	17 (70.8%)	7 (29.2%)	
*Place to conduct online teaching*			
Home	48 (63.2%)	28 (36.8%)	.400
Work place	10 (52.6%)	9 (47.4%)	

### Factors affecting students’ online education satisfaction

Table 5 shows students’ assessment of the factors influencing their satisfaction on online education, and 40 factors were found to be significant ones (*P* < 0.05). After comparing the mean ratings of each question from satisfied students and dissatisfied students, 9 factors were identified as negative factors for online education satisfaction and 31 factors were positive factors. Based on the ranking of the factors’ mean value, the severity of the COVID-19 situation (Sa-01), the absence of experimental/practical classes (Sd-06), the uncertainty of the university-opening date and the following teaching arrangement (Sa-05), the severity of economic issues related to the COVID-19 (Sa-06) and the lockdown due to the COVID-19 (Sa-02) were the top 5 barriers to students’ online education. However, the top 5 factors that were influential in facilitating success in their online learning included: well-accomplished assignments (Sb-10), adequate frequency to access the internet for online learning (Sb-11), adequate support and help from the university during the online learning process (Sd-10), adequate self-discipline (Sb-05) and adequate use of the course resources (Sb-13).

**Table 5:** Students’ assessment of factors influencing the online education satisfaction

Code	Questions about the factors influencing the online education satisfaction	Comparison of positive and negative responses to the factors		
		All students (*n* = 230)	Students inside China (*n* = 77)	Students outside China (*n* = 153)
	*Crisis-related category*	−	/	−
Sa-01	How much do the epidemiological data related to the COVID-19 pandemic affect your online learning?	−	/	−
Sa-02	How much does the lockdown due to the COVID-19 pandemic affect your learning state?	−	/	−
Sa-03	How much are you satisfied with your current learning environment?	+	/	+
Sa-04	How well are you adapt to the sudden change of the teaching pattern (from fully offline to fully online)?	+	+	+
Sa-05	How much are you affected by the uncertainty of the university-opening date and the following teaching arrangement?	−	/	−
Sa-06	How much do the economic issues related to the COVID-19 affect your online learning?	−	/	−
	*Learner-related category*	+	/	+
Sb-01	How much do you enjoy independent study?	+	+	/
Sb-02	How much are you concerned about the data cost for the online learning?	−	/	−
Sb-03	How much are you familiar with the online learning technologies?	/	/	/
Sb-04	How much do you enjoy having the flexibility to regulate your time for online learning?	+	/	+
Sb-05	How much do you consider yourself self-disciplined?	+	/	+
Sb-06	How much are you motivated for the online learning?	+	/	+
Sb-07	How much are you affected by the sense of distance during your learning?	−	−	−
Sb-08	How much do you participate in the discussions?	+	/	+
Sb-09	How much do you like to post messages while having live e-lectures?	+	/	+
Sb-10	How well do you accomplish your assignments?	+	/	/
Sb-11	How frequently do you access the Internet for online learning?	+	/	+
Sb-12	How frequently do you review the online courses?	+	/	+
Sb-13	How much do you use the course resources (such as preview and review materials) provided?	+	/	+
Sb-14	How much are you satisfied with your performance in the quizzes?	+	+	+
Sb-15	How much are you affected by the limited contacts with your classmates during the learning process?	/	/	/
	*Instructor-related category*	+	+	+
Sc-01	How would you evaluate the teachers’ overall performance?	+	/	+
Sc-02	How adequate is the duration of the online courses?	+	+	+
Sc-03	How adequate are attention and care you have received from the teachers during the learning process?	+	/	+
Sc-04	How adequate are the interactions with the teachers during online teaching process?	+	/	+
Sc-05	How adequate is the time scheduled for discussions (such as Q and A class)?	+	/	+
Sc-06	How helpful are the course resources (such as preview and review materials) provided by the teachers?	+	+	+
Sc-07	How easy is the use of the course resources?	+	+	+
Sc-08	How easy is it for you to communicate with the teachers via the communication tool?	+	/	+
Sc-09	How available are the teachers apart from the scheduled communication time?	+	/	+
Sc-10	How clearly are the teaching contents expressed?	+	+	+
	*Course-related category*	+	+	+
Sd-01	How well the online courses are administrated?	+	/	+
Sd-02	How effective are the design and arrangement of the online courses?	+	+	+
Sd-03	How much is your online learning affected by the network conditions?	−	/	−
Sd-04	How much is your online learning affected by the jet lag in your country?	/	/	−
Sd-05	How much is your online learning affected by the facility you are using?	−	/	−
Sd-06	How much is your online learning affected by the absence of experimental/practical classes?	−	/	/
Sd-07	How much helpful is the playback support in the online courses?	+	+	/
Sd-08	How much are you satisfied with the tools for discussions (Q and A class)?	+	+	+
Sd-09	How much are you satisfied with the assignments specifically oriented to online learning?	+	+	+
Sd-10	How adequate do you think the support and help from the university you have received during your online learning process?	+	+	+
Sd-11	How clear are the objectives for the online courses?	+	+	+
Sd-12	How much collaborative learning is there during the online learning?	+	/	+

1. Code of the questions: The letter S refers to the questions for students; the letters a, b, c and d represent 4 categories (crisis-related category, learner-related category, instructor-related category and course-related category), respectively

2. Students’ ratings of the questions did not comply with the normal distribution, so Kruskal–Wallis rank-sum test was performed to compare if there was any significant difference between the students who were satisfied with the online education and those who were not satisfied in each question

3. The symbol “/” indicated that there was no significant difference between the satisfied students and the dissatisfied students

4. The symbol “ + ” indicated that there was significant difference between the satisfied students and the dissatisfied students, and the factor was positively related with the satisfaction of online education (the mean rating of satisfied students was higher than that of dissatisfied students)

5. The symbol “−” indicated that there was significant difference between the satisfied students and the dissatisfied students, and the factor was negatively related with the satisfaction of online education (the mean rating of dissatisfied students was higher than that of satisfied students)

Furthermore, the influential factors were quite different for students inside China and those outside China (Table 5). The top 5 beneficial factors for students inside China were the clarity of the online course objective (Sd-11), the playback support for the online courses (Sd-07), the capability of independent study (Sb-01), the quality of the course resources (Sc-06) and the easy use of the course resources (Sc-07). As for students outside China, the top 5 promoters were adequate frequency to access the Internet for online learning (Sb-11), adequate support and help from school during the online learning process (Sd-10), adequate self-discipline (Sb-05), adequate use of the course resources (Sb-13) and good administration of the online courses (Sd-01).

While there were no negative factors found to affect students inside China, the top 5 obstacles for students outside China were the severity of the COVID-19 situation (Sa-01), the uncertainty of university-opening date and following teaching arrangement (Sa-05), the sense of distance (Sb-07), the lockdown (Sa-02) and the severity of economic issues related to COVID-19 (Sa-06).

### Factors affecting teachers’ online education satisfaction

Table 6 shows teachers’ assessment of factors influencing their satisfaction with online education, and 17 factors were found to be significant ones (*P* < 0.05). Among them, 5 were negative factors—the sense of distance (Tb-10), the lockdown (Ta-02), the severity of the COVID-19 situation (Ta-01), the stressful workload for online teaching (Ta-05), and the absence of experimental/practical classes (Td-05). In the 12 success factors, the top 5 were: good administration of the online courses (Td-01), effective design and arrangement of the online courses (Td-02), good teaching environment (Ta-03), satisfying outcomes of students’ quizzes (Tc-06) and satisfying tools for discussions (Td-10).

**Table 6:** Teachers’ assessment of factors influencing the online education satisfaction

Code	Questions about the factors influencing the online education satisfaction	Comparison of positive and negative responses to the factors (*n* = 95)
	*Crisis-related category*	−
Ta-01	How much do the epidemiological data related to the COVID-19 pandemic affect your online teaching?	−
Ta-02	How much does the lockdown due to the COVID-19 pandemic affect your teaching state?	−
Ta-03	How much are you satisfied with your current teaching environment?	+
Ta-04	How well are you adapt to the sudden change of the teaching pattern?	/
Ta-05	How much stress do you feel from the online teaching workload due to the crisis?	−
	*Instructor-related category*	/
Tb-01	How stressed do you feel due to English as the instruction medium for the online courses?	/
Tb-02	How much does the shortage of communications with your colleagues affect your teaching?	/
Tb-03	How much are you familiar with the online teaching technologies?	/
Tb-04	How much does the thought of the possibility that a wider range of audience other from your students may observe your teaching process affect your teaching?	/
Tb-05	How positive are you that you pay adequate attention to the students?	+
Tb-06	How much do you consider you are passionate during the online class?	+
Tb-07	How much do you think you achieve the teaching objectives via the online instruction?	+
Tb-08	How stressed do you feel about the extra requirements catering to the online teaching?	/
Tb-09	How many materials can you provide to the students?	/
Tb-10	How much are you affected by the sense of distance during your teaching?	−
Tb-11	How much pressure do you feel when teaching directly to international students in the classroom?	/
Tb-12	How stressed are you now that you are teaching online in English without having to face the students directly?	/
Tb-13	How adequate are you prepared for online teaching?	/
	*Learner-related category*	+
Tc-01	How would you evaluate the students’ overall performance?	+
Tc-02	How would you rate students’ assignments?	+
Tc-03	How active are the students during the online teaching process and interactive discussions?	+
Tc-04	How cooperative are the students during their online learning?	+
Tc-05	How adequate is the time for discussions with students?	/
Tc-06	How much are you satisfied with students’ performance in the quizzes?	+
	*Course-related category*	/
Td-01	How well the online courses are administrated?	+
Td-02	How effective are the design and arrangement of the online courses?	+
Td-03	How much have you been affected by the network conditions?	/
Td-04	How much is your online teaching affected by rearranged course schedule to cope with the jet lag in students’ countries?	/
Td-05	How much does the absence of experimental/practical classes affect your teaching the theoretical knowledge?	−
Td-06	How much are you satisfied with the live streaming platforms for online education?	/
Td-07	How much is your online teaching affected by the facility you are using?	/
Td-08	How much is your online teaching affected by the absence of the blackboard?	/
Td-09	How much helpful is the playback support in the online courses?	/
Td-10	How much are you satisfied with the tools for discussions (Q and A class)?	+
Td-11	How adequate do you think the support and help from the university you have received during your online teaching process?	/
Td-12	How often are you affected by the operational problems?	/
Td-13	How much is your teaching affected by tight time to prepare online teaching?	/
Td-14	How much collaborative learning do you arrange during your online teaching process?	/
Td-15	How adequate is the technical support you have received for conducting the online courses?	/

1. Code of the questions: The letter T refers to the questions for teachers; the letters a, b, c and d represent 4 categories (crisis-related category, learner-related category, instructor-related category and course-related category), respectively

2. Teachers’ ratings of the questions did not comply with the normal distribution, so Kruskal–Wallis rank-sum test was performed to compare if there was any significant difference between the teachers who were satisfied with the online education and those who were not satisfied in each question

3. The symbol “/” indicated that there was no significant difference between the satisfied teachers and the dissatisfied teachers

4. The symbol “ + ” indicated that there was significant difference between the satisfied teachers and the dissatisfied teachers, and the factor was positively related with the satisfaction of online education (the mean rating of satisfied teachers was higher than that of dissatisfied teachers)

5. The symbol “−” indicated that there was significant difference between the satisfied teachers and the dissatisfied teachers, and the factor was negatively related with the satisfaction of online education (the mean rating of dissatisfied teachers was higher than that of satisfied teachers)

### Crisis-, learner-, instructor- and course-related categories affecting online education satisfaction

All the factors were classified into 4 major categories according to their attributes, namely crisis-, learner-, instructor- and course-related categories. A binary logistic regression model was performed for multiple-factor analysis to determine the association of the related categories with the online education satisfaction (see Table 7). For all the students, their online education satisfaction was closely associated with the crisis and the instructors (*P* < 0.01). Moreover, factors associated with the crises (*P* < 0.05), instructor (*P* < 0.01) and course (*P* < 0.05) significantly affected students outside China, which was not the case for students inside China. And for teachers, their online education satisfaction was associated with the crisis and the learners (*P* < 0.01).

**Table 7:** Crisis-, learner-, instructor- and course-related categories affecting online education satisfaction

Categories	All students		Students inside China		Students outside China		Teachers	
	Coefficients	*p*-value	Coefficients	*p*-value	Coefficients	*p*-value	Coefficients	*p*-value
Crisis-related	− 0.695	.009	/		− 0.685	.035	− 1.644	.001
Learner-related	0.586	.133	/		0.779	.108	1.966	.000
Instructor-related	1.324	.000	.833	.123	2.196	.000	/	
Course-related	0.094	.801	1.214	.087	− 1.230	.024	/	

The symbol “/” indicated that the particular category did not significantly affect the particular population’s attitude towards the online education, so the related data were excluded from the binary logistic regression model for multiple-factor analysis

### Experiences and expectations of online education, students versus teachers

Table 8 compares students’ and teachers’ opinions regarding their experiences and expectations of online education. Significant differences were found in their responses of the online education perceptions and expectations for the future delivery of education courses. Specifically, the percentage of the teachers who were satisfied with the online education effect (61.1%) was higher than that of the satisfied students (36.5%). After pairwise comparison, it was found that a higher ratio of teachers preferred blended education (53.7%) compared to the students (30.9%), while a higher proportion of students preferred face-to-face education (58.7%) than teachers (35.8%).

**Table 8:** Comparison of experiences of current online education and expectations of future online education, students VS teachers

Experiences and expectations of online education	Students (*n* = 230)	Teachers (*n* = 95)	*p*-value
*Attitude towards online education effect*			
Satisfied	84 (36.5%)	58 (61.1%)	.000
Not satisfied	146 (63.5%)	37 (38.9%)	
*Time and dedication required by the online learning*			
More than it should require	132 (57.4%)	58 (61.1%)	.611
Neither more or less	92 (40.0%)	36 (37.9%)	
Less than it should require	6 (2.6%)	1 (1.0%)	
*Expectations for future delivery of education courses*			
Face-to-face delivery method	135 (58.7%)	34 (35.8%)	.000
Online delivery method	24 (10.4%)	10 (10.5%)	
Blended delivery method	71 (30.9%)	51 (53.7%)	

## Discussion

In the study, we conducted a comprehensive assessment of the online education among international medical and nursing students from LMICs and their teachers during the COVID-19 crisis, and we found that: (i) most teachers were satisfied with the online education experiences, whereas most students were dissatisfied; (ii) teachers’ professional title, students’ year of study, continent of origin and location of current residence significantly affected the online education satisfaction; (iii) the most influential facilitating factor to online education for students was a well-accomplished course assignment while that it was good administration of the online courses for teachers; (iv) the most influential barrier to online education for students was the severity of the COVID-19 situation while it was the sense of distance for teachers.

Currently, around 75% of the international students are staying outside China during the pandemic, and this percentage tends to continue rising as international students still keep leaving China; meanwhile, it is unpredictable when the out-China students could return to the campus [25]. Therefore, it seems that the large number of out-China students will study and even graduate remotely over a longer period of time. In this aspect, our study provides important references for government and medical schools in China to improve their online health sciences education for international students, whose training qualities will affect the healthcare delivery in their future practice locations around the world.

### Effectiveness of online education

The effectiveness of online learning in imparting a health sciences curriculum has been supported by some empirical evidence, exemplified by medical and nursing students’ promising outcomes in tests [12, 26] as well as their positive perceptions on the acquisition of knowledge [27, 28] and skills [29, 30]. Several recent studies also reported a high overall satisfaction rate for the online education during the COVID-19 crisis among Chinese (80.29%) and Indian (97.14%) students and teachers at medical schools [14, 31]; however, a study from Jordan reported a low satisfaction rate (26.77%) with the distance e-learning amid the pandemic among medical students in clinical years [13]. In this study, we found a comparatively low satisfaction rate in international students and a medium satisfaction rate in their teachers, whose online education experiences were significantly influenced by various factors.

### Factors affecting online education

#### Factors related to the crisis

The crisis was noted to significantly reduce both students’ and teachers’ education satisfaction. Although the pandemic intensely affected the students staying outside China, it did not affect the students staying inside China, which might be explained by the different pandemic circumstances in different regions.

During our survey period, the data of the existing infected cases in China had declined remarkably, while in many other parts of the world it was on the rise [32]. At the time of the survey, 153 (66.5%) of the students in our study were staying overseas, among whom, 102 (44.3%) were in India, where the newly diagnosed cases were generally above 1000 each day [32]. It is therefore understandable that the factors related to the COVID-19 pandemic—the epidemiological data (Sa-01), the lockdown (Sa-02), future uncertainties (Sa-05) and the economic issues (Sa-06) exerted a detrimental impact on online learning for students outside China, while these factors did not affect students staying in China. As the pandemic in different countries still fluctuates, medical schools in China should keep an eye on the pandemic in students’ current place of residence and pay extra attention to those faced with more serious situations.

On the other side, the teachers, who were also staying in China and thus supposed to be free from the influence of the crisis, turned out to be greatly influenced. The reasons might be two fold. First, 46.3% of the teachers in our study teach clinical subjects, who work in the clinical setting and were exposed to the high-risk environment during the COVID-19 crisis, suffering from stresses such as long working hours, risk of infection, physical fatigue, and so on [33], which presented challenges to their online teaching conditions. Second, a massive number of courses had been transitioned from offline to online due to the crisis. As for the 74.7% of the teachers without previous online teaching experience, they were forced to bear huge pressures according to the wholly online requirement; as for rest of the experienced teachers, they could hardly fulfil their expectations with online teaching as a suboptimal mode with such short notice, considering that the previously built online courses were well prepared with plenty of time.

Among the crisis-related factors, the current learning environment (Sa-03) and the current teaching environment (Ta-03) were found to promote the education experiences for students outside China and teachers inside China, who share something in common—they are studying or teaching at a familiar and relaxing environment, surrounded by their family or friends. According to Zhang et al. [11], a comfortable environment and support from close ones can help overcome negative emotions during the pandemic, so students and teachers in more familiar surroundings are more likely to cope with adverse feelings better and thus enjoy their online education more. This inference is reinforced by the finding in our study that the learning environment was not a promoter for students inside China, who are far away from home and familial relationships. This fact implies that in-China students’ emotional stresses, such as homesickness and loneliness, which might become more serious as the separation with family prolongs, need to be pacified with the necessary timely psychological support.

#### Factors related to learners and instructors

In this study, the international students’ learning satisfaction was closely associated with the influence from the teachers rather than themselves. Interestingly, the teachers’ education satisfaction was, in fact, closely associated with the influence from the students.

The results showed that factors related to the attitude and engagement of learners (self-discipline, cooperation, learning motivation, time regulation, assignment completion, access frequency to the internet, participation in discussions, message postings, lesson reviewing, and resource usages) and instructors (attention to the students, interaction with students, resource preparation, availability for students, passion for teaching) significantly affected online education. These findings have also been emphasised in the literature as important aspects influencing the effectiveness of online learning [10, 23, 34]. When taking a closer look, we found that many of the above-mentioned learning attitude and engagement factors affected only students outside China but not students inside China. This could be attributed to the fact that many students staying overseas largely depend on the asynchronised mode of online learning due to the time change or network issues, which, therefore, demands higher autonomy and initiative. However, as there is a change regarding the availability of some online live streaming platforms and online chatting tools in some international students’ countries of origin during the past year, there has been a massive decline in the proportion of synchronised classes while the asynchronised mode now serves as the mainstream to deliver online courses to and conduct real-time interactions with students to facilitate the accessibility of the coursework. Predictively, students’ learning attitude is likely to have a stronger impact on their online learning outcome, so administrators and teachers are encouraged to make every effort to maintain students’ engagement.

Familiarity of technological skills was not found to be an influential factor for both students and teachers, manifested in their rating results for items Sb-03 and Tb-03, which is in line with Bao’s viewpoint [35] that learning attitudes outweigh technology skills regarding online learning. Bernardo et al. [23] also stresses that students’ quick adaptation in using the Internet for academic studies can compensate for their unfamiliarity with the web technologies at the beginning. However, some researchers conclude a two-way causal relationship between technological skills and students’ engagement in online learning, emphasising that the mastery of the former strengthens the latter [34], and the latter facilitates the development of the former [36].

#### Factors related to courses

Our results showed that technical issues, such as network conditions (Sd-03) and facilities used (Sd-05), significantly affected students’ dissatisfaction of online learning. This finding is in agreement with studies in India [28], Malaysia [37], Ghana [38] and South Africa [39]. All the students in our study were learning online in LMICs. As documented by Frehywot et al. [12], the implementation of e-learning among health sciences students in the resource-constrained LMICs is usually technically challenged, and the main problems include slow speed of accessing and downloading from the Internet, poor quality images/sounds, limited electronic facilities and frequent electrical power failures. To ameliorate technical disturbance on e-learning in less privileged settings, scholars propose the application of approaches requiring less bandwidth, such as a hybrid teaching method or a digital library independent of the Internet [40]. During the past year, XZMU has adopted several measures to optimise the online education for the international students in technically challenged LMICs. For example, to reduce the negative effect of poor Internet connectivity and prohibitive data cost, teachers are recommended to upload short videos instead of longer ones. Furthermore, the online assessment methods have also been adjusted from closed-end questions at fixed examination hours to open-ended questions, clinical case analyses or essays within a more flexible time frame to ensure fairness in case some students suffered from issues related to the network or electronic power.

Additionally, the absence of experimental/practical classes was considered as an important inhibiting factor for online medical and nursing education by both students and teachers. Our results also showed that students in their clinical years were significantly more dissatisfied than those in their basic years, which implies that the unavailability of the clinical experience possibly produces a more adverse effect on theory learning than the inability to undertake experiments does. In fact, thanks to digital technology, experimental operations traditionally learned in laboratories and clinical skills traditionally acquired in hospitals are presently allowed for online learning. For example, some medical schools in Australia and the UK have already replaced dissection with e-textbooks and online resources in anatomy teaching [41]. Moreover, during the COVID-19 pandemic, an American institution utilised telehealth clinics for surgical residents to maintain their clinical exposure, enabling the trainees to partake in the whole process electronically, such as discussing with the attending surgeon over the phone after gathering the patient’ medical record, videoconferencing with the medical team and the patient to formulate the treatment plan [42]. Although it seems feasible to perform experiments and clinical practice online, which provides an example of how to cope with the current policy of restricting gatherings, it is argued that face-to-face contact and interactions with classmates as well as instructors are preferred for experimental hours [43] and the hands-on clinical operative experiences and direct patient care are indispensable [42]. Therefore, there is an urgent need for universities in China to cope with these challenges creatively by establishing collaborations not only internationally but also domestically. In a recent national conference pertaining to international medical education, proposals have been put forward that medical schools in China are recommended to collaborate collectively to connect with overseas medical schools and hospitals especially those situated in the IMSs’ and INSs’ countries of origin, so as to provide alternative experiences locally for the experimental classes and clinical practice they are missing [44].

### Expectation of online education

Despite the fact that online learning is well accepted as a sound and enjoyable method, health sciences students hardly see it as a replacement for offline learning [45]. Instead, e-learning is more regarded as a supplement to support traditional didactic teaching [30] or a component of blended learning [28]. In our study, we found that students’ acceptance of solely online learning during the pandemic was low, but nearly half of the students expressed interest in online learning as at least part of the future education style. Additionally, nearly two-thirds of the teachers (64.2%) voted for online education for their future teaching. The future preference for online/blended learning is even higher among medical students or teachers from Jordan (80.7%) and Saudi Arabia (88.0%) [13, 15]. A year has passed since our survey and the learning trajectories for in-China students have basically returned to normal with all the face-to-face experimental classes and clinical practice fully resumed. Meanwhile, not surprisingly, online learning has been retained by many medical schools as an important component to supply offline teaching of the theoretical knowledge, which mirrors the expectations of students and teachers from our survey.

Despite the massive disruptions the pandemic has brought to the medical and nursing education for international students, this technological revolution also opens the door to a new world. Not only online learning, but online assessment and online graduation are also born at this moment, which helps medical schools speed up the process of internationalisation and explore worldwide distance education possibilities.

## Conclusions

This study has identified the key factors which have influenced the satisfaction of international medical and nursing students and their teachers towards their online education in China during the pandemic. This information can be used to inform medical schools on how to improve their online teaching delivery in a similar crisis in the future and enhance their education outcome. The current COVID-19 crisis significantly affects the online education outcome of international students and teachers, whereas the influence might be in disparity according to the pandemic situation at their current places of residence in different countries. Medical schools are advised to take care of in-China international students’ psychological status and create a more enjoyable learning environment for them, while also taking care of out-China international students who are studying from a far distance where they may be subject to the pressures from slow Internet connection and high data cost. In addition, it is suggested that universities in China need to collaborate collectively to connect with overseas medical schools and hospitals, so as to create practice opportunities for international students.

### Limitations

The study may be more representative if this is a cross-institutional study with a larger sample size. Furthermore, the impact of participants’ decisions to return home or remain in China for the winter break on their subsequent satisfaction was not analysed in the study. In addition, although it has been explained ahead of the focus group discussion as well as at the beginning of the questionnaire that this study was just for the research and improvement of online education quality, there might be some students and teachers who did not provide true ratings due to certain considerations.

## Data Availability

The datasets used and/or analysed during the current study are available from the corresponding author on reasonable request.

## Abbreviations

COVID-19: Coronavirus disease 2019
UNESCO: United Nations Educational, Scientific and Cultural Organization
IMS: International medical student
INS: International nursing student
LMIC: Low- and middle-income country

## References

[1] Daniel SJ (2020). Education and the COVID-19 pandemic. Prospects.

[2] UNESCO. COVID-19 educational disruption and response. 2020. https://en.unesco.org/covid19/educationresponse. Accessed 23 Nov 2020.

[3] Ministry of Education of the People’s Republic of China. Instructions of online learning in general institutes of higher education in China during the COVID-19 crisis. 2020. http://www.moe.gov.cn/srcsite/A08/s7056/202002/t20200205_418138.html Accessed 18 Apr 2020.

[4] Ministry of Education of the People’s Republic of China. Notice about the “nonstop learning” in elementary and secondary schools in China during the school extension period. 2020. http://www.moe.gov.cn/srcsite/A06/s3321/202002/t20200212_420435.html Accessed 20 Apr 2020.

[5] Li G. Challenges confronted by international medical student education in China and counterplans. Qingdao: 2019 Academic Annual Conference of International Medical Student Education in China, International Medical Education Branch of China Education Association for International Exchange; 2019.

[6] Li W, Liu C, Liu S, Zhang X, Shi R, Jiang H (2020). Perceptions of education quality and influence of language barrier: graduation survey of international medical students at four universities in China. BMC Med Educ.

[7] Low & middle income. The World Bank, Washington. 2020. https://data.worldbank.org/income-level/low-and-middle-income Accessed 16 Dec 2020.

[8] Shi R, Lian G, Liu L, Ding D, Liu X, Huang Y (2020). Teaching methods of paediatrics for international student during the prevention and control period of COVID-19. Med Educ Res Pract..

[9] McCutcheon K, Lohan M, Traynor M (2014). Martin D (2014) A systematic review evaluating the impact of online or blended learning vs face-to-face learning of clinical skills in undergraduate nurse education. J Adv Nurs..

[10] Pei L, Wu H (2019). Does online learning work better than offline learning in undergraduate medical education? A systematic review and meta-analysis. Med Educ Online.

[11] Zhang X, Jia W, Duan L (2020). An investigation and analysis of psychological status of 1486 medical students in the period of COVID-19. J Inner Mongolia Med Univ.

[12] Frehywot S, Vovides Y, Talib Z, Mikhail N, Ross H, Wohltjen H (2013). E-learning in medical education in resource constrained low- and middle-income countries. Hum Resour Health.

[13] Al-Balas M, Al-Balas HI, Jaber HM, Obeidat K, Al-Balas H, Aborajooh EA (2020). Distance learning in clinical medical education amid COVID-19 pandemic in Jordan: current situation, challenges, and perspectives. BMC Med Educ.

[14] Rathee N, Sarkar C. COVID-19 Lockdown: how the pandemic bringing change in Indian education system. In: Impact of COVID-19 and pandemic lockdown in India. Balewadi: Eureka Publications; 2020.

[15] Rajab MH, Gazal AM, Alkattan K (2020). Challenges to online medical education during the COVID-19 pandemic. Cureus..

[16] Qin L, Zhang Y, Xue M, Xu B, Wang L, Zhang J (2020). Nursing students’ perceptions towards online learning during the COVID-19 pandemic. Health Vocat Educ.

[17] Central People's Government of the People’s Republic of China. How to arrange course studies and exams for international students enrolled in China’s institutions, who have trouble coming back to school? 2020. http://www.gov.cn/xinwen/2020-03/31/content_5497687.htm Accessed 30 Apr 2020.

[18] Hampton D, Culp-Roche A, Hensley A, Wilson J, Otts JA, Thaxton-Wiggins A (2020). Self-efficacy and satisfaction with teaching in online courses. Nurs Educ.

[19] Johnson SD, Aragon SR, Shaik N, Palma-Rivas N (2000). Comparative analysis of learner satisfaction and learning outcomes in online and face-to-face learning environment. J Interact Learn Res.

[20] Stickney LT, Bento RF, Aggarwal A, Adlakha V (2019). Online higher education: faculty satisfaction and its antecedents. J Manag Educ.

[21] Alqurashi E (2019). Predicting student satisfaction and perceived learning within online learning environments. Distance Educ.

[22] Rogers ME, Creed PA, Searle J (2009). The development and initial validation of social cognitive career theory instruments to measure choice of medical specialty and practice location. J Career Assess.

[23] Bernardo V, Ramos MP, Plapler H, Figueiredo LFP, Nader HB, Anção MS (2004). Web-based learning in undergraduate medical education: development and assessment of an online course on experimental surgery. Int J Med Inform.

[24] Zheng B, Lin C, Kwon JB (2020). The impact of learner-, instructor-, and course-level factors on online learning. Comput Educ..

[25] Liu X. Challenges in post-pandemic era. Beijing: Online conference of educational quality assurance for China-educated international medical students, International Medical Education Branch of China Education Association for International Exchange; 2020.

[26] Gerdsprasert S, Pruksacheva T, Panijpan B, Ruenwongsa P (2011). An interactive web-based learning unit to facilitate and improve intrapartum nursing care of nursing students. Nurse Educ Today..

[27] Montayre J, Sparks T (2018). As I haven’t seen a T-cell, video-streaming helps: nursing students’ preference towards online learning materials for biosciences. Collegian.

[28] Shenoy SJ, Kuriakose C (2016). Effects of E-learning as a teaching learning method in medical education. J Evol Med Dent Sci.

[29] McMullan M, Jones R, Lea S (2011). The effect of an interactive e-drug calculations package on nursing students’ drug calculation ability and self-efficacy. Int J Med Inform.

[30] Warnecke E, Pearson S (2011). Medical students’ perceptions of using e-learning to enhance the acquisition of consulting skills. Australas Med J.

[31] Kan D, Jing Z, Yang H, Sun W, Liu X, Gao X (2020). Research and analysis of online learning of clinical theory course for medical undergraduates. China Med Educ Tech.

[32] World Health Organization. Coronavirus disease (COVID-19) Weekly epidemiological update and weekly operational update. 2020. https://www.who.int/emergencies/diseases/novel-coronavirus-2019/situation-reports Accessed 28 Nov 2020.

[33] Rajkumar RP (2020). COVID-19 and mental health: a review of the existing literature. Asian J Psychiatr..

[34] Muilenburg LY, Berge ZL (2005). Student barriers to online learning: a factor analytic study. Distance Educ.

[35] Bao W (2020). COVID-19 and online teaching in higher education: a case study of Peking University. Hum Behav & Emerg Tech.

[36] Robinson CC, Hullinger H (2008). New benchmarks in higher education: student engagement in online learning. J Educ Bus.

[37] Seluakumaran K, Fedelis JF, Ismail R, Husain R (2011). Integrating an open-source course management system (Moodle) into the teaching of a first-year medical physiology course: a case study. Adv Physiol Educ.

[38] Adanu RMK, Adu-Sarkodie Y, Opare-Sem O, Nkyekyer K, Donkor P, Lawson A (2010). Electronic learning and open educational resources in the health sciences in Ghana. Ghana Med J.

[39] Mars M (2012). Building the capacity to build capacity in e-health in sub-Saharan Africa: the KwaZulu-Natal experience. Telemed J & E-Health.

[40] Missen C (2005). Internet in a box: the eGranary digital library serves scholars lacking internet bandwidth. New Rev Inf Networking.

[41] Patel SB, Mauro D, Fenn J, Sharkey DR, Jones C (2015). Is dissection the only way to learn anatomy? Thoughts from students at a non-dissecting based medical school. Perspect Med Educ.

[42] Chick RC, Clifton GT, Kaitlin M, Peace KM, Propper BW, Hale DF (2020). Using technology to maintain the education of residents during the COVID-19 pandemic. J Surg Educ.

[43] Franchi T (2020). The impact of the COVID-19 pandemic on current anatomy education and future careers: a student’s perspective. Anat Sci Educ.

[44] Board members. Workshop on international medical education in China during the pandemic. The Sixth Session of its First Conference of the Enlarged Board Committee. International Medical Education Branch of China Education Association for International Exchange; 2020.

[45] Huynh R (2017). The role of E-Learning in medical education. Acad Med.

